# Objective Assessment of Tooth Mobility Using the Osstell Device: A Pilot Study

**DOI:** 10.3390/diagnostics16081126

**Published:** 2026-04-09

**Authors:** Kübra Erdoğan Eryıldız, Fariz Selimli, Ahmet Can Haskan, Osman Fatih Arpağ

**Affiliations:** 1 Specialist Dentist, İstanbul 34890, Türkiye; 2 Department of Oral and Maxillofacial Surgery, Faculty of Dentistry, Hatay Mustafa Kemal University, Antakya 31001, Türkiye; 3 Department of Periodontology, Faculty of Dentistry, Hatay Mustafa Kemal University, Antakya 31001, Türkiye

**Keywords:** periodontal index, tooth mobility, resonance frequency analysis, periotest

## Abstract

**Background/Objectives**: The objective assessment of natural tooth mobility remains challenging in clinical practice. This pilot study aimed to investigate the feasibility, repeatability, and agreement of a modified implant stability measurement system adapted for natural teeth using a custom-fabricated titanium bracket and a modified SmartPeg. **Methods**: Sixteen systemically healthy patients (10 males, six females) and 94 single-rooted permanent teeth with varying mobility grades were included. The tooth mobility was assessed using the Miller Mobility Index, Periotest M, and resonance frequency analysis (RFA) with the Osstell Beacon device. For the Osstell measurements, a custom titanium bracket bonded to the buccal tooth surface allowed for the placement of a modified SmartPeg. Each tooth was measured twice under standardized conditions, and mean values were recorded. The statistical analyses included Spearman correlation analysis, Cohen’s kappa for agreement with Miller categories, and intraclass correlation coefficients (ICCs) to assess the measurement repeatability. **Results**: The mean Periotest value was 12.70 ± 13.69, and the mean ISQ (implant stability quotient) value was 69.45 ± 19.37. The repeated measurements demonstrated excellent intra-examiner repeatability for both devices (ICC > 0.95). The Periotest values showed substantial agreement with the Miller mobility grades (κ = 0.763; *p* < 0.001), whereas the Osstell values demonstrated weak agreement with these ordinal categories (κ = 0.094; *p* = 0.048). A strong negative correlation was observed between the Periotest and Osstell measurements irrespective of the scales (r = −0.865; *p* < 0.001). **Conclusions**: In natural dentition, the resonance frequency analysis demonstrated reproducible measurements under controlled experimental conditions and showed measurable associations with conventional mobility assessments. However, the method remains investigational. The findings do not establish clinical validity for the routine assessment of natural tooth mobility. Further studies with larger sample sizes and statistical models accounting for patient-level clustering are required before clinical implementation can be considered. This study is registered at ClinicalTrials.gov (NCT07188168).

## 1. Introduction

Tooth mobility refers to the physiological or pathological movement capability of a tooth within the alveolar bone [1]. Under normal conditions, teeth exhibit limited movement due to the presence of the periodontal ligament. However, factors such as trauma, periodontal disease, occlusal trauma, or loss of supporting structures can increase this mobility [2]. Tooth mobility is a significant indicator of periodontal health and plays a crucial role in diagnosis, treatment planning, and monitoring treatment outcomes [3]. Traditionally, tooth mobility is subjectively measured by the Miller Mobility Index [4]. This method involves applying buccolingual force to the tooth using the ends of two dental instruments and observing the extent of movement. Grading systems, such as the Miller Mobility Index, are often used for this purpose. Although simple and widely used, this method is subjective, and the results may vary between clinicians. Therefore, in recent years, there has been increasing interest in developing more objective and quantitative techniques for evaluating tooth stability [1,5,6].

The most commonly used techniques include the Periotest and resonance frequency analysis [7,8]. The Periotest, first introduced in the late 1980s, is a diagnostic device designed to objectively evaluate the mobility of natural teeth and dental implants by quantifying the damping characteristics of the periodontium or peri-implant tissues [9,10]. The device uses an electromagnetically controlled tapping rod to deliver a series of short, controlled mechanical impulses (typically 16 per measurement) to the surface of the tooth. The contact time between the probe and the tooth is then recorded, which reflects the stiffness and damping properties of the surrounding tissues [11]. The system provides numerical values, known as the Periotest value (PTV), enabling more standardized and reproducible measurements compared with manual methods [12]. Nevertheless, variations have been reported in the measurements obtained with the Periotest device, both between different examiners and within the same examiner when evaluating different regions. These discrepancies may be related to factors such as the angle of application and the need to maintain a specific distance between the device and the tooth during measurement [13].

Originally developed for implant stability evaluation, the Osstell device measures the resonance frequency of a transducer attached to a structure and provides results in terms of implant stability quotient (ISQ) values [14]. Resonance frequency analysis (RFA) has been reported to be a reliable, reproducible, and objective method, particularly because of its well-established use in implantology [15]. Although several studies have demonstrated a linear correlation between the Periotest and RFA measurements on dental implants [10,16], comparative analyses have also suggested that Osstell may offer more sensitive and precise measurements than the Periotest device [11,17].

Over the years, various electronic devices have been introduced to provide a more objective assessment of tooth mobility. Despite the availability of numerous techniques and instruments, their reliability in accurately quantifying tooth mobility remains limited and somewhat controversial. One of the main challenges is the complex viscoelastic behavior of the periodontal ligament, which significantly influences tooth displacement and is difficult to reproduce precisely under controlled loading conditions in laboratory settings. Furthermore, tooth mobility is influenced by both physiological and pathological factors, and the mobility ranges may vary among different tooth types and between individuals. This variability complicates the establishment of clear and standardized criteria for the tooth mobility assessment [1]. Therefore, this pilot study aimed to investigate the applicability of the Osstell device for assessing the mobility of natural teeth with different mobility grades and to evaluate the correlation between its measurements and those obtained from conventional clinical mobility assessment methods.

## 2. Materials and Methods

This study was conducted at the Department of Oral and Maxillofacial Surgery, Faculty of Dentistry, Hatay Mustafa Kemal University. The ethical approval was obtained from the Ethics Committee of Hatay Mustafa Kemal University (Approval No: 2022/35). This study was conducted in accordance with the ethical principles of the Declaration of Helsinki.

Patient Selection

After obtaining ethical approval, all the eligible participants were informed in detail about the study procedures, and their written informed consent was obtained prior to their participation. A total of 16 patients (10 males and six females) were included in the study. Mobility measurements were performed on 94 permanent teeth, including 40 teeth with an intact periodontium and 54 teeth affected by periodontal disease. These teeth exhibited varying degrees of mobility, allowing for the evaluation of the compatibility and accuracy of the measurement methods across a wide range of mobility grades.

The patients presenting during the recruitment period were screened according to the predefined inclusion and exclusion criteria. If a patient did not meet the inclusion criteria, the next eligible patient was enrolled.

The inclusion criteria were as follows: the individuals must be aged between 18 and 45 years; the patients must be systemically healthy; the individuals must have completed non-surgical periodontal therapy at least 3 months prior to enrollment and be enrolled in a periodontal maintenance program; there must be an absence of bleeding when probing; and single-rooted teeth must be present.

The exclusion criteria included a history of psychiatric or neurological disorders; impaired muscle coordination or neuromuscular dysfunction; ongoing orthodontic treatment or recent dental trauma affecting tooth stability; presence of parafunctional habits such as bruxism or clenching; and tooth-related pathologies, including endodontic lesions or root resorption.

Clinical Examination

The periodontal probing depth was measured during the clinical examination at six sites per tooth (mesiobuccal, mid-buccal, distobuccal, mesiolingual, mid-lingual, and distolingual). The mean probing depth per tooth was calculated in millimeters. The measurements were performed using a UNC-15 periodontal probe (UNC-15, Hu-Friedy, Chicago, IL, USA).

Mobility Measurement

All the measurements were performed twice for each tooth by the same calibrated examiner to ensure consistency and to eliminate inter-examiner variability. The repeated measurements were conducted under identical conditions for each assessment method (Miller mobility grading, Periotest M, and Osstell Beacon). A 5 min interval was maintained between the measurements obtained with different devices in order to minimize the potential transient periodontal ligament fluid displacement effects.

Miller Mobility Measurement

**Figure 1:**
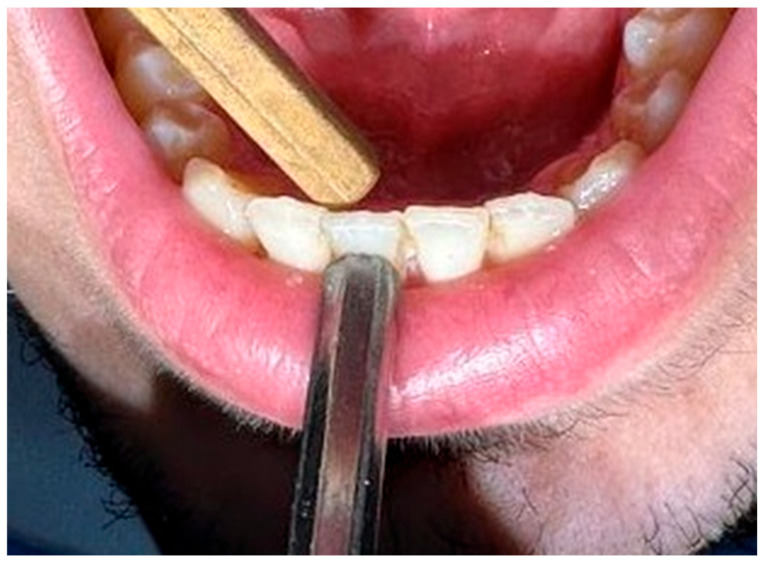
The clinical assessment of tooth mobility using Miller’s classification. The tooth mobility is evaluated by applying pressure with two instrument handles on the labial and lingual surfaces of a mandibular anterior tooth.

Tooth mobility was assessed according to Miller’s classification by evaluating the buccolingual tooth displacement using the handles of two dental instruments (Figure 1).

Miller Mobility Grades:

Grade 0: Physiological mobility; no bone loss and probing depth approximately 2–3 mm.

Grade I: Slight mobility; tooth movement ≤1 mm horizontal (buccolingual) direction

Grade II: Moderate mobility; tooth movement >1 mm but ≤2 mm in the horizontal direction.

Grade III: Severe mobility; tooth movement >2 mm horizontally and/or the presence of vertical mobility (i.e., depressible in the socket) [4].

Periotest Device Measurement:

Measurement Protocol

**Figure 2:**
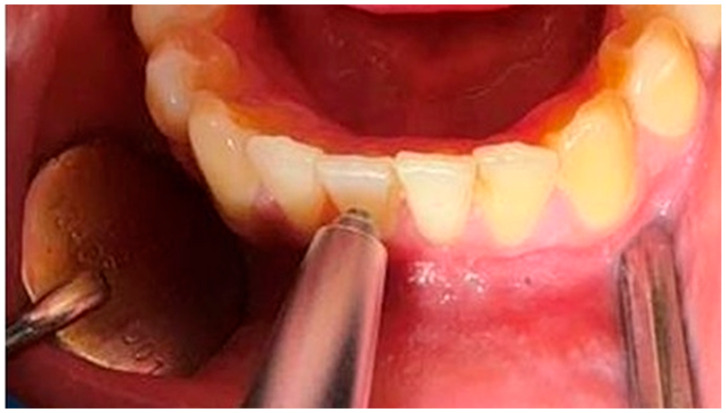
The objective measurement of tooth mobility using the Periotest device, applied to a mandibular anterior tooth.

Prior to each session, the device was calibrated according to the manufacturer’s instructions. The target tooth was thoroughly cleaned to remove plaque, saliva, and blood. The Periotest probe was positioned perpendicular (90°) to the long axis of the tooth. For the measurements, the device was positioned approximately 1–2 mm away from the mid-buccal surface and oriented perpendicular to the long axis of the tooth. (Figure 2). Two consecutive measurements were obtained from each tooth, and the mean of those readings was recorded as the PTV [18].

Interpretation of Periotest Values [19]

PTV Range

−8 to +9: clinically firm teeth

+10 to +19: palpable mobility

+20 to +29: visible mobility

+30 to +50: severe mobility under pressure from the lip or tongue.

Resonance Frequency Analysis

**Figure 3:**
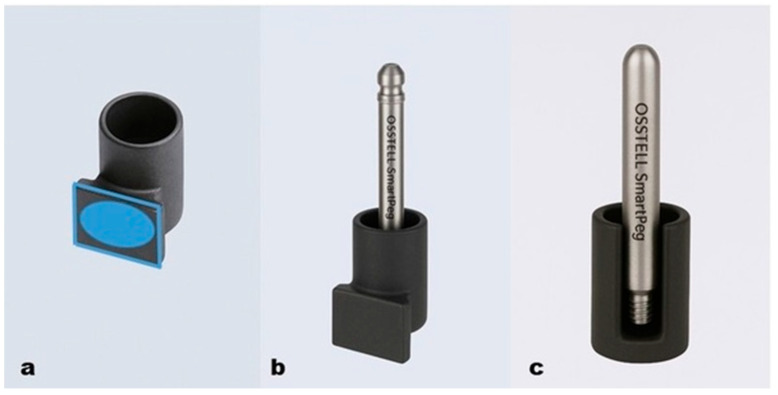
The titanium bracket, developed for measuring tooth mobility with the Osstell device, is shown. (**a**) The cylindrical component with a flat surface, indicated by a blue elliptical ring, designed to be bonded to the tooth. (**b**) The external view of the modified Smartpeg seated within the cylindrical component. (**c**) The internal view of the modified Smartpeg engagement within the cylindrical component.

To assess tooth stability, RFA was performed using the Osstell Beacon device (Osstell, AB, Gothenburg, Sweden). A custom-fabricated titanium bracket was employed to accommodate the modified SmartPeg and ensure the standardized placement on each tooth. The bracket was carefully aligned with the long axis of the tooth and positioned apically (Figure 3a).

Bracket Specifications:

• Material: Medical-grade titanium, precision-machined using CNC technology.
• Design: Cylindrical base with a centrally located, threaded slot for modified SmartPeg insertion.

Modified SmartPeg Thread Slot:

• Internally threaded to enable clockwise insertion of the modified SmartPeg (Figure 3b,c).
• Micro-threaded, tapered, and centered aperture.
• Diameter: approximately 1.2–1.5 mm.

Tooth Contact Surface:

• Flat base designed to conform to the buccal surface of the tooth.
• Smooth texture with micro-mechanical retention properties (Figure 3a).

**Figure 4:**
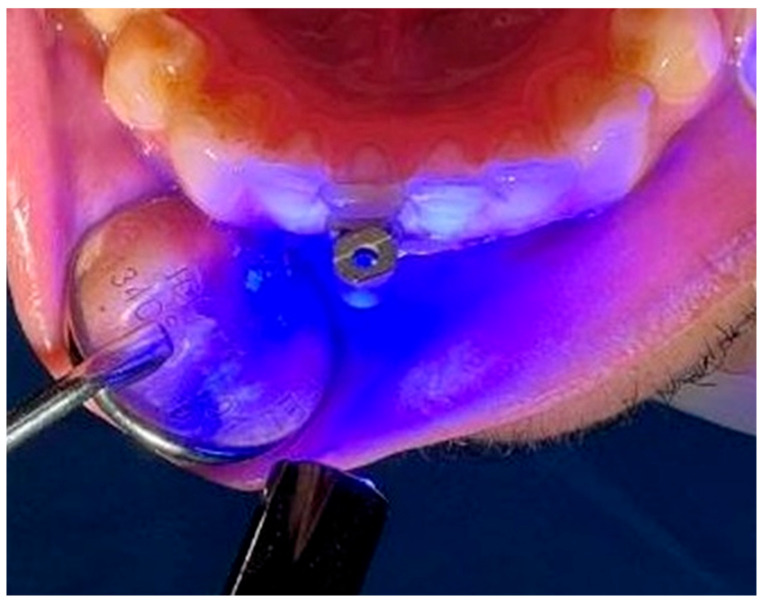
The bonding of the titanium bracket to the tooth and the light polymerization.

**Figure 5:**
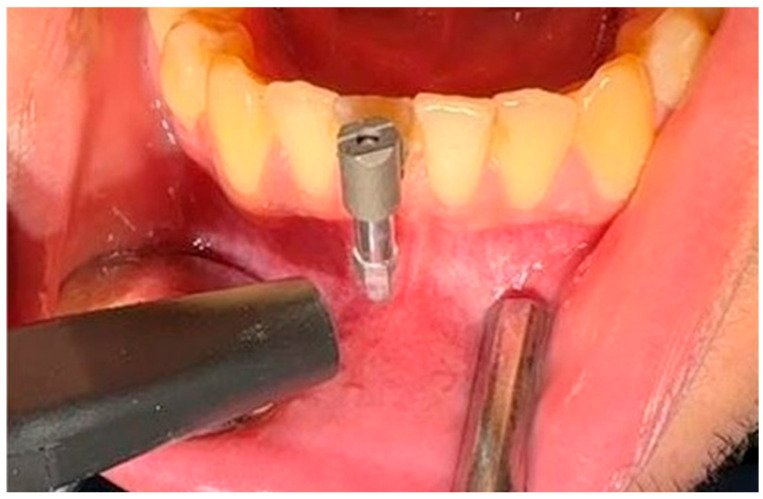
The determination of tooth stability using the Osstell device. Note that the intraoral placement of the modified Smartpeg is aligned parallel to the root axis.

Osstell Measurement Protocol:

The bracket apparatus was placed on the buccal surface of the respective tooth using a flowable composite (Figure 4). To minimize methodological variability due to custom bracket fabrication and bonding, all the brackets were made from the same material, with standardized dimensions, and bonded using a uniform adhesive protocol. All bonding procedures were performed by a single operator to ensure consistency in the adhesive thickness and bracket positioning. Care was taken to orient the slot designated for the modified SmartPeg toward the root (Figure 5).

The modified SmartPeg was then manually tightened (finger-tight, approximately 4–6 Ncm). Two consecutive RFA measurements were obtained from the buccal aspect, which was perpendicular to the long axis of the root. The mean of these readings was recorded as the final RFA value (ISQ) for each tooth. During the measurement, the Osstell device was held perpendicular (90°) to the modified SmartPeg at a distance of 2–5 mm from the buccal surface. The ISQ unit, ranging from 0 to 100, reflects the stiffness at the implant–bone interface, with higher values indicating greater stability [20].

Statistical Analysis

The data distribution was assessed using the Shapiro–Wilk test. The relationships between the numerical variables were evaluated with Spearman’s correlation coefficient. The agreement between the mobility classes and the Periotest/Osstell measurements was assessed using Cohen’s Kappa statistic, which was interpreted as follows: values <0.00 = poor, 0.00–0.20 = slight, 0.21–0.40 = fair, 0.41–0.60 = moderate, 0.61–0.80 = substantial, and 0.81–1.00 = almost perfect agreement. The reliability of the stability scores that were obtained from both devices was evaluated using the intraclass correlation coefficient (ICC) based on a two-way mixed-effects model with a single measurement (average measure) approach. An ICC ≥0.75 was considered indicative of satisfactory reliability. The intra-examiner reliability was assessed through the agreement of two consecutive measurements. A high intra-examiner agreement was considered indicative of the measurement reproducibility.

All the statistical analyses were performed using SPSS (Statistical Package for the Social Sciences, version 29.0), with its significance set at *p* < 0.05.

## 3. Results

A total of 16 patients (62.5% male, *n* = 10; 37.5% female, *n* = 6) and 94 teeth were included. The most frequently evaluated teeth were 41 (11.7%) and 11, 12, 21, 31, and 32 (each 9.6%), while the least frequent were 44 (1.1%) and 34 (2.1%) (Table 1).

Using the Periotest device, the overall mean PTV was 12.70 ± 13.69 (median: 8.25; range: –2.75 to 50). For the RFA, the overall mean ISQ value was 69.45 ± 19.37 (median: 77; range: 20–90).

The inter-examiner reliability for the Miller mobility grades, assessed via the ICC (two-way random-effects and absolute agreement), showed excellent agreement (ICC = 0.96), indicating high consistency and reproducibility.

The mean probing depths, according to the Miller mobility grades, were as follows: Grade 0 = 1.86 mm (*n* = 40), Grade I = 1.94 mm (*n* = 30), Grade II = 2.71 mm (*n* = 13), and Grade III = 4.47 mm (*n* = 11). The overall mean probing depth for all teeth was 2.31 mm.

The agreement between two consecutive measurements was high for both devices. The Periotest’s first and second attempts yielded means of 12.63 ± 13.75 (median: 7.80; –2.90 to 50) and 12.77 ± 13.70 (median: 8.45; –2.60 to 50), respectively (ICC = 0.996; 95% CI: 0.994–0.997; *p* < 0.001). The first and second attempts of the Osstell yielded means of 69.04 ± 19.61 (median: 77; 20–90) and 69.86 ± 19.18 (median: 77; 20–90), respectively (ICC = 0.998; 95% CI: 0.997–0.999; *p* < 0.001). The ICC values ≥0.95 indicate an almost perfect level of reliability for both methods (Table 2).

The correlations between the measurements obtained with the Periotest and Osstell devices across the Miller mobility grades were evaluated (Table 3). According to Miller’s classification, the mean Periotest values increased progressively from grade 0 to grade III, while Osstell values decreased. The Periotest measurements were 1.86 ± 2.84 (median 1.78; min–max: −2.75–8.05) in grade 0, 11.94 ± 5.16 (11.75; 1.75–20.7) in grade I, 23.21 ± 5.61 (24.5; 12.95–34.4) in grade II, and 41.71 ± 7.50 (38.10; 32.65–50) in grade III. In contrast, the Osstell values were 82.04 ± 4.97 (80; 74–90) in grade 0, 73.50 ± 7.28 (76.25; 60.5–88.5) in grade I, 59.46 ± 11.66 (61; 32.5–76) in grade II, and 24.45 ± 6.08 (23; 20–42) in grade III. The intraclass correlation coefficient (ICC) values between the two measurement methods were 0.002 (95% CI: 0.001–0.008) for grade 0, 0.015 (0.012–0.018) for grade I, 0.055 (0.032–0.166) for grade II, and 0.115 (0.037–0.268) for grade III. The corresponding *p*-values were 0.998, 0.999, 0.960, and 0.936, respectively.

No significant correlation was found between the values obtained from the Osstell and Periotest measurements when classified according to the Miller mobility grades (Table 3).

Table 4 presents the distribution of the Periotest and Osstell measurements according to the Miller mobility grades and the level of agreement between these device-based scales and the clinical classification. For the Periotest scale, values between –8 and +9 were observed exclusively in Miller Grade 0 teeth (100%). The values in the +10 to +19 range were predominantly associated with Grade I mobility (63.3%), whereas +20 to +29 values were mainly observed in Grade II teeth (69.2%). The +30 to +50 range corresponded entirely to the Grade III mobility (100%). Overall, a substantial and statistically significant agreement was found between the Periotest scale and Miller’s classification (κ = 0.763; 95% CI: 0.655–0.871; *p* < 0.001).

The mean PTV increased progressively with an increasing mobility grade. The mean PTV was 1.86 ± 2.84 in Grade 0, 11.94 ± 5.16 in Grade I, 23.21 ± 5.61 in Grade II, and 41.71 ± 7.50 in Grade III, indicating a clear trend of the Periotest values increasing with higher levels of tooth mobility.

For the Osstell scale, the RFA values greater than 69 were observed exclusively in Grade 0 teeth (100%), while values between 60 and 69 were mainly distributed between Grade I (40.0%) and Grade II (46.1%). The values below 60 were primarily associated with Grade II (38.5%) and Grade III (100%) mobility. In contrast to the Periotest scale, the agreement between the Osstell scale and Miller’s classification was weak but statistically significant (κ = 0.094; 95% CI: 0.089–0.099; *p* = 0.048). The mean RFA values decreased as the mobility increased, with the mean values of 82.04 ± 4.97 for Grade 0, 73.50 ± 7.28 for Grade I, 59.46 ± 11.66 for Grade II, and 24.45 ± 6.08 for Grade III. These findings indicate that higher tooth mobility is associated with increasing Periotest values and decreasing Osstell values. Overall, the Periotest measurements demonstrated a stronger level of agreement with Miller’s clinical mobility classification than with the Osstell scale.

Table 5 presents the distribution of the Periotest scale categories across the Osstell measurement ranges and the agreement between the two device-based methods. Teeth that had Periotest values between –8 and +9 were exclusively associated with the Osstell values that were greater than 69 (100%), indicating high stability measurements in both systems. In the +10 to +19 Periotest range, the Osstell values were distributed between 60 and 69 (54.5%) and >69 (45.5%), suggesting a moderate level of stability.

For the Periotest values between +20 and +29, the Osstell measurements were mainly observed in the 60–69 range (54.5%), while 36.4% of these teeth had Osstell values <60, indicating a reduced stability. In the highest Periotest category (+30 to +50), all teeth (100%) presented Osstell values <60, corresponding to the lowest stability levels.

Overall, 63.8% of the teeth showed Osstell values >69, 19.1% were within the 60–69 range, and 17.0% had values <60. The agreement between the Periotest and Osstell categorical scales was weak but statistically significant (κ = 0.088; 95% CI: 0–0.194; *p* = 0.045), indicating that although the two methods follow a generally similar trend in stability assessment, their categorical classifications show limited concordance.

**Figure 6:**
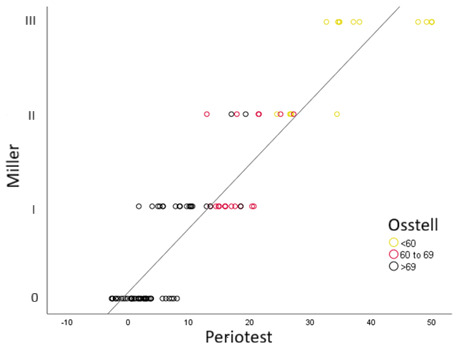
A scatter plot of Miller grades and Periotest and Osstell values.

A strong positive correlation was observed between the Miller mobility scores and the Periotest values in cases with Osstell values <60 (r = 0.777; *p* < 0.001). For cases with Osstell values between 60 and 69, a moderate, statistically significant positive correlation was found between the Miller grades and the Periotest values (r = 0.477; *p* = 0.045). Similarly, in cases where the Osstell values were >69, a strong positive correlation was again observed between the Miller grades and the Periotest values (r = 0.725; *p* < 0.001) (Figure 6).

**Figure 7:**
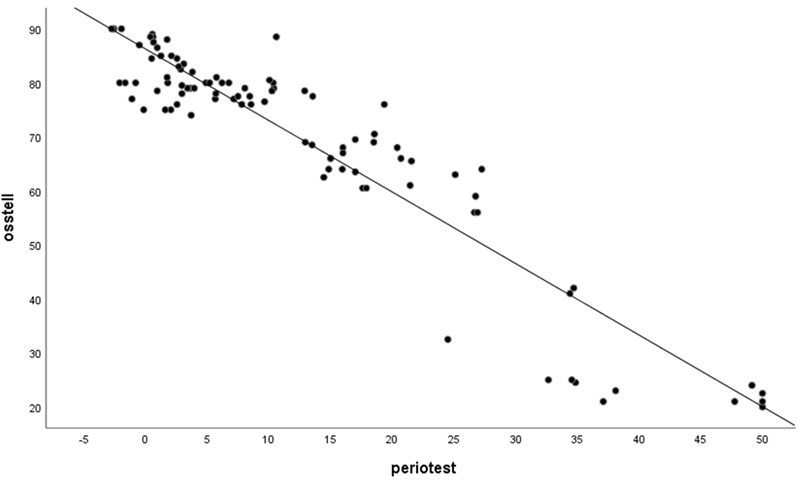
A Scatter plot of the Osstell and Periotest values.

A very strong negative correlation was detected between the mean Osstell and Periotest values irrespective of the scales of the two methods (r = –0.865; *p* < 0.001). The scatter plot illustrating the relationship between these two variables is presented in Figure 7.

## 4. Discussion

Tooth mobility is an important parameter for assessing the prognosis of teeth [1]. A distinction exists between physiological mobility and pathological mobility, and classifying tooth mobility accordingly is an essential part of the diagnostic process [21]. In clinical practice, mobility is most easily assessed using the Miller Mobility Index [4]. However, subjective assessment may complicate diagnostic and prognostic decision-making, especially in borderline cases. Consequently, numerous studies have demonstrated that the Periotest device can be applied in periodontology, implantology, orthodontics, and traumatology [13,22,23]. The Periotest measures both horizontal and vertical tooth mobility and provides reproducible results [24,25]. The device operates by converting the contact time between the tooth and a percussive probe into a numerical value using a series of impacts at 0.2 m/s with a force of 8 g. As a result, the readings are sensitive to changes in the fluid resistance of the periodontal tissues [26]. Some studies have suggested that stabilizing devices may be necessary during measurement to reduce extraneous movement [27,28]. Due to the Periotest method presenting certain difficulties in evaluating the periodontal tissue conditions, it has been widely preferred in order to measure implant stability [29,30]. Consequently, alternative techniques, such as laser systems, intraoral scanners, or contact-free vibration devices, have been proposed for measuring the viscosity, elastic modulus, and frequency characteristics of periodontal tissues [1].

In the present study, a custom apparatus was developed to attach a modified Smartpeg to the buccal surfaces of permanent human teeth using composite resin. Using this setup, tooth mobility was evaluated via Osstell resonance frequency analysis. To our knowledge, this represents the first study comparing the RFA of natural teeth with established subjective and objective assessments. The RFA allows for the determination of stiffness or displacement at the implant-bone interface [31,32]. The method involves attaching a modified Smartpeg to the implant, with the device probe positioned close to the sensor in bucco-lingual and mesio-distal directions during the emission of electromagnetic pulses [33]. When combined with radiography or ultrasonography, RFA provides a comprehensive assessment of both the mechanical and biological aspects of implant integration [34].

Some studies suggest that the Periotest may be more reliable for intraoperative measurements [35], whereas others report lower reproducibility and sensitivity compared with RFA [36]. RFA has been shown to offer higher sensitivity and specificity in implant stability assessments [22]. The Periotest is advantageous in clinical practice due to its lower cost and ease of use, but careful stabilization, positioning, and maintenance of proper distance are critical to reliable measurements [17]. Deviations at this stage may reduce the reliability of measurements.

The Periotest values correspond to Miller grades according to the manufacturer: –8 to +9 = Grade 0, +10 to +19 = Grade I, +20 to +29 = Grade II, and +30 to +50 = Grade III [37]. For the Osstell measurements, ISQ values <60 indicate a low stability, 60–69 indicate a moderate stability, and ≥70 or greater represent a high stability [38]. In implantology, an insertion torque ≥30 Ncm with ISQ >60 is recommended for loading, whereas values <20 Ncm are associated with a higher risk of failure [39,40,41]. In this study, the ISQ values obtained from natural teeth were categorized based on these thresholds [42].

Although the comparison is theoretical, Miller’s classification and the Periotest and Osstell values demonstrated partial compatibility. The strongest agreement was observed between the Miller grades and Periotest values. The agreement between the Periotest and Osstell was statistically significant but weak. For example, among 40 teeth with Osstell values of ≥70, the Periotest values ranged between –8 and +9, whereas in 11 teeth with the same Osstell value, the Periotest values ranged from +10 to +29. This discrepancy explains the weak agreement observed between the Periotest and Osstell values in natural teeth. Since the Periotest scale adapted to Miller’s classification may not be universally accepted, Osstell values may lead to incomplete scientific interpretations. Previous studies have also reported that the Periotest measurements may not be a valid parameter for assessing periodontal damage in teeth with different periodontal conditions, and histological studies are required for this purpose [43]. This may be explained by the fact that the damping characteristics of each tooth are unique and may vary according to age and sex [44]. This important consideration relates to the statistical comparison between the clinical Miller mobility classification and the instrument-based measurements. The Miller mobility represents an ordinal clinical scale, whereas Periotest and Osstell provide continuous numerical outputs reflecting the biomechanical characteristics of the tooth–periodontal complex. In the present study, continuous measurements were categorized to allow for a comparison using Cohen’s kappa analysis. However, such categorization inevitably reduces the information contained in the continuous data and may partly influence the level of agreement observed, particularly for the Osstell measurements. Moreover, the grouping of the Osstell values was based on thresholds that are commonly used in the implant stability literature. Because natural teeth possess a periodontal ligament and therefore exhibit different biomechanical behavior compared with ankylosed dental implants, these thresholds may not accurately reflect the mobility categories in natural teeth. Consequently, the relatively low agreement observed between the Osstell values and Miller’s classification should be interpreted cautiously.

In the present study, a statistically significant agreement was observed between consecutive measurements obtained with both the Periotest and Osstell devices. Also, a strong negative correlation was observed between the mean Periotest and Osstell values, meaning that as the Periotest values increased, the Osstell values decreased. These findings indicate that mobility measurements obtained with both devices are reproducible and reliable in natural teeth. However, despite these findings, it should be noted that all measurements were performed on anterior and premolar teeth, which greatly facilitated the positioning of both devices. This limitation may affect the precision of the measurements and the generalizability of the findings.

The root morphology and periodontal support influence the mechanical behavior and viscoelastic damping [45]. The RFA depends on the boundary conditions and structural stiffness. While widely used for implant stability assessment, the RFA behavior in teeth with periodontal ligament support may differ due to viscoelastic characteristics [46]. To reduce variability, the study was limited to single-rooted anterior teeth, and the experimental model should not be interpreted as a routine clinical method. The study also has several limitations, including the relatively small sample size and the requirement to attach a custom-fabricated component to the tooth during the mobility measurement.

Orthodontic bracket debonding procedures may lead to alterations in the enamel surface and the formation of adhesive remnants depending on the technique used. Previous studies have reported that the removal of orthodontic brackets and subsequent adhesive clean-up procedures may affect the surface morphology and roughness of dental tissues. For instance, Ghaleb et al. demonstrated that different debonding and clean-up systems can produce varying degrees of enamel surface alteration and residual adhesive, potentially influencing the surface integrity. Similarly, Moradi et al. reported that the debonding process and adhesive removal procedures may increase surface roughness and cause topographic changes on dental materials and enamel surfaces. These findings suggest that mechanical interventions performed during bracket removal can lead to microstructural surface alterations, highlighting the importance of using minimally invasive debonding and polishing techniques in clinical practice [47,48]. If roughened areas remain untreated, they may promote dental plaque accumulation, which can subsequently lead to enamel demineralization and the development of caries and periodontal inflammation [49]. A finite element analysis study has shown that certain debonding techniques—such as shearing off, compression, lateral torque, and frontal torque—can generate elevated stress values in the enamel, periodontal ligament, and surrounding bone. Among these techniques, compression has been reported to be the gentlest approach for the periodontal ligament and alveolar bone, whereas lateral torque should be applied with particular caution in teeth with periodontal tissue loss, as it may concentrate stress within the compromised supporting structures. However, it should also be noted that in those experimental studies, all bracket bonding procedures were performed using conventional acid-etching and bonding protocols, which may influence the magnitude and distribution of stresses generated during the debonding process [50]. In addition, patients may experience varying levels of pain and discomfort during orthodontic bracket removal. Several authors have investigated the factors associated with this discomfort and reported that variables such as sex, tooth type, arch side, and the presence of dental restorations show only a weak association with the level of discomfort experienced. Among these variables, tooth type may have a relatively greater influence on patient discomfort. Two main factors that may affect discomfort during bracket debonding are tooth mobility and the direction of the applied force. Intrusive forces are generally better tolerated because the periodontal structures are anatomically organized to resist masticatory forces [51].

Concerns may arise regarding clinical applicability and measurement stability when an additional apparatus is bonded to mobile teeth. Because complete standardization during bracket positioning and bonding may be difficult to achieve, some variability in the measurement results may occur. However, all bonding procedures were carried out by the same researcher and followed minimally invasive principles to minimize enamel damage. In addition, unlike the conventional acid-etch bonding protocol commonly used in classical orthodontic bracket systems, only a bonding application was used in the present study. The bracket was removed from the tooth surface using a bracket-removing plier without the application of additional force. Following the removal, no visible residue remained on the tooth surface. Accordingly, the procedure performed on the tooth surface would not be expected to result in detectable damage.

## 5. Conclusions

These findings suggest a relationship between the measurement methods; however, the results should be interpreted with caution because multiple teeth were obtained from a limited number of patients. A total of 94 teeth were obtained from only 16 patients, meaning that several teeth from the same individual were included in the analysis. As teeth within the same patient may share biological and biomechanical characteristics, these observations may not be fully independent. It should also be emphasized that the present study represents an early feasibility investigation.

Within these limitations, a relationship between the Miller grades and the Periotest values was observed, whereas attempts to align this relationship—originally validated for natural teeth—with the Osstell measurements, which are primarily used for implant stability assessment, may partly explain the observed statistical incongruence. However, when analyzed independently of categorical scales, a strong negative correlation between the Periotest and Osstell values was identified. This finding suggests that the Osstell measurements may reflect stability changes in natural teeth under the experimental conditions of the present study.

Although the mean Osstell measurements were reproducible and demonstrated a strong inverse relationship with the mean Periotest values in this experimental setting, these findings do not establish clinical validity for the routine assessment of natural tooth mobility. Future studies involving larger and more diverse samples, repeated measurements, and statistical models that account for patient-level clustering (e.g., mixed-effects models) are needed to more robustly evaluate the biological safety and practical applicability of this approach and to determine clinically meaningful thresholds for natural teeth.

## Figures and Tables

**Table 1:** The demographic profile and tooth distribution of the participants.

	*n* (%)
Male	10 (62.5)
Female	6 (37.5)
Tooth number (FDI)	
11	9 (9.6)
12	9 (9.6)
13	7 (7.4)
21	9 (9.6)
22	8 (8.5)
23	7 (7.4)
31	9 (9.6)
32	9 (9.6)
33	3 (3.2)
34	2 (2.1)
41	11 (11.7)
42	7 (7.4)
43	3 (3.2)
44	1 (1.1)

*n* = number of teeth (*n* = 94). Tooth identification (shown in the left column) was performed according to the two-digit tooth numbering system of the FDI World Dental Federation.

**Table 2:** The agreement between the first and second measurements obtained by the Periotest and Osstell devices.

	First Attempt Mean ± SD/ M [Min–Max]	Second Attempt Mean ± SD/ M [Min–Max]	ICC (95%CI)	*p*
Periotest	12.63 ± 13.75/ 7.8 [−2.9 to +50]	12.77 ± 13.7/ 8.45 [−2.6 to +50]	0.996 (0.994–0.997)	<0.001
Osstell (Ncm)	69.04 ± 19.61/ 77 [20–90]	69.86 ± 19.18/ 77 [20–90]	0.998 (0.997–0.999)	<0.001

M: median. CI: coefficient interval. ICC: intraclass correlation coefficient. *p*-value reflects the statistical significance of the ICC. Ncm: newton/centimeter.

**Table 3:** The correlation of the Periotest and Osstell values by Miller’s classification.

Miller/Grades	0 Mean ± SD/ M [Min–Max]	I Mean ± SD/ M [Min–Max]	II Mean ± SD/ M [Min–Max]	III Mean ± SD/ M [Min–Max]
Periotest	1.86 ± 2.84/ 1.78 [−2.75–8.05]	11.94 ± 5.16/ 11.75 [1.75–20.7]	23.21 ± 5.61/ 24.5 [12.95–34.4]	41.71 ± 7.50/ 38.10 [32.65–50]
Osstell	82.04 ± 4.97/ 80 [74–90]	73.50 ± 7.28/ 76.25 [60.5–88.5]	59.46 ± 11.66/ 61 [32.5–76]	24.45 ± 6.08/ 23 [20–42]
ICC	0.002 (0.001–0.008)	0.015 (0.012–0.018)	0.055 (0.032–0.166)	0.115 (0.037–0.268)
*p*	0.998	0.999	0.960	0.936

M: median. ICC: intraclass correlation coefficient. *p*-value reflects the statistical significance of the ICC.

**Table 4:** The distribution and scale-level correlations of the Periotest and Osstell values according to Miller’s classification.

	Miller Mobility Grades					
	Grade 0 *n* (%)	Grade I *n* (%)	Grade II *n* (%)	Grade III *n* (%)	κ (95%CI)	*p*
Periotest Scale						
−8 to +9	40 (100.0)	9 (30.0)	0 (0.0)	0 (0.0)	0.763 (0.655–0.871)	<0.001 *
+10 to +19	0 (0.0)	19 (63.3)	3 (23.1)	0 (0.0)		
+20 to +29	0 (0.0)	2 (6.7)	9 (69.2)	0 (0.0)		
+30 to +50	0 (0.0)	0 (0.0)	1 (7.7)	11 (100.0)		
PTV						
mean ± SD/ M [min–max]	1.86 ± 2.84/ 1.78 [–2.75–8.05]	11.94 ± 5.16/ 11.75 [1.75–20.70]	23.21 ± 5.61/ 24.50 [12.95–34.40]	41.71 ± 7.50/ 38.10 [32.65–50]		
%95 CI	0.96–2.77	10.02–13.87	19.82–26.60	36.68–46.75		
Osstell Scale					0.094 (0.089–0.099)	0.048 *
<60	0 (0.0)	0 (0.0)	5 (38.5)	11 (100.0)		
60 to 69	0 (0.0)	12 (40.0)	6 (46.1)	0 (0.0)		
>69	40 (100.0)	18 (60.0)	2 (15.4)	0 (0.0)		
RFA						
mean ± SD/ M [min–max]	82.04 ± 4.97/ 80 [74–90]	73.50 ± 7.28/ 76.25 [60.50–88.50]	59.46 ± 11.66/ 61 [32.50–76]	24.45 ± 6.08/ 23 [20–42]		
	
%95 CI	80.45–83.63	70.78–76.22	52.42–66.50	20.37–28.54		

The *κ* coefficient was derived from Cohen’s Kappa statistic. M: median. CI: coefficient interval. RFA: resonance frequency analysis. PTV: Periotest value. n: number of teeth. * indicates statistical significance.

**Table 5:** The correlations between the Periotest and Osstell values.

Osstell Scale		−8 to +9 *n* (%)	+10 to +19 *n* (%)	+20 to +29 *n* (%)	+30 to +50 *n* (%)	Overall *n* (%)	κ (95% CΙ)	*p*
<60		0 (0.0)	0 (0.0)	4 (36.4)	12 (100.0)	16 (17.0)	0.088 (0–0.194)	0.045 *
60 to 69		0 (0.0)	12 (54.5)	6 (54.5)	0 (0.0)	18 (19.1)		
>69		49 (100.0)	10 (45.5)	1 (9.1)	0 (0.0)	60 (63.8)		
Overall		49 (100.0)	22 (100.0)	11 (100.0)	12 (100.0)	94 (100.0)		

The *κ* coefficient was derived from Cohen’s Kappa statistic. M: median. CI: coefficient interval. n: number of teeth. * indicates statistical significance.

## Data Availability

The raw data supporting the conclusions of this article will be made available by the authors on request.

## Abbreviations

The following abbreviations are used in this manuscript:

ICC	Intraclass correlation coefficient
ISQ	Implant stability quotient
PTV	Periotest value
RFA	Resonance frequency analysis
